# Co-Circulation of Chikungunya and Multiple DENV Serotypes and Genotypes, Western Indonesia 2015–2016

**DOI:** 10.3390/v14010099

**Published:** 2022-01-06

**Authors:** Harapan Harapan, Alice Michie, Timo Ernst, Kritu Panta, Mudatsir Mudatsir, Benediktus Yohan, Sotianingsih Haryanto, Suzi McCarthy, R. Tedjo Sasmono, Allison Imrie

**Affiliations:** 1 Medical Research Unit, School of Medicine, Universitas Syiah Kuala, Banda Aceh 23111, Indonesia; harapan@unsyiah.ac.id (H.H.); mudatsir@unsyiah.ac.id (M.M.); 2 Tropical Disease Centre, School of Medicine, Universitas Syiah Kuala, Banda Aceh 23111, Indonesia; 3 Department of Microbiology, School of Medicine, Universitas Syiah Kuala, Banda Aceh 23111, Indonesia; 4 School of Biomedical Sciences, University of Western Australia, Nedlands, WA 6009, Australia; alice.michie@uwa.edu.au (A.M.); timo.ernst@uwa.edu.au (T.E.); kritu.panta@uwa.edu.au (K.P.); suzi.mccarthy@health.wa.gov.au (S.M.); 5 Eijkman Institute for Molecular Biology, Jakarta 10430, Indonesia; yohan@eijkman.go.id (B.Y.); sasmono@eijkman.go.id (R.T.S.); 6 Faculty of Medicine and Health Science, Universitas Jambi, Jambi 36361, Indonesia; sotianingsih@yahoo.com; 7 Raden Mattaher Hospital, Jambi 36361, Indonesia; 8 Pathwest Laboratory Medicine, Nedlands, WA 6009, Australia

**Keywords:** dengue, chikungunya, epidemiology, phylogenetic, Aceh, Jambi, Indonesia

## Abstract

Dengue is a mosquito-borne disease of public health concern affecting tropical and subtropical countries, including Indonesia. Although studies on dengue epidemiology have been undertaken in Indonesia, data are lacking in many areas of the country. The aim of this study was to determine dengue virus (DENV) and chikungunya virus (CHIKV) molecular epidemiology in western regions of the Indonesian archipelago. A one-year prospective study was conducted in Aceh and Jambi in 2015 and 2016, respectively, where patients with dengue-like illness were enrolled. Of 205 patients recruited, 29 and 27 were confirmed with dengue in Aceh and Jambi, respectively, and three from Jambi were confirmed with chikungunya. DENV-1 was the predominant serotype identified in Aceh while DENV-2 was predominant in Jambi. All DENV-1 and DENV-2 from both regions were classified as Genotype I and Cosmopolitan genotype, respectively, and all DENV-3 viruses from Jambi were Genotype I. Some viruses, in particular DENV-1, displayed a distinct lineage distribution, where two DENV-1 lineages from Aceh were more closely related to viruses from China instead of Jambi highlighting the role of travel and flight patterns on DENV transmission in the region. DENV-2 from both Aceh and Jambi and DENV-3 from Jambi were all closely related to Indonesian local strains. All three CHIKV belonged to Asian genotype and clustered closely with Indonesian CHIKV strains including those previously circulating in Jambi in 2015, confirming continuous and sustainable transmission of CHIKV in the region. The study results emphasize the importance of continuous epidemiological surveillance of arboviruses in Indonesia and simultaneous testing for CHIKV among dengue-suspected patients.

## 1. Introduction

Dengue, caused by infection with any of four dengue virus (DENV-1 to DENV-4) serotypes, is a major international public health concern and is associated with significant morbidity, mortality, and economic losses [1,2,3]. Nearly 60% of the Indonesian population (277 million) live in areas where DENV activity is known. Currently, Indonesia reports the highest average number of dengue cases in Asia [4,5]. The incidence rate (IR) of dengue hemorrhagic fever (DHF) has had a cyclical pattern, with peaks occurring approximately every 6–8 years [6,7]. Although the annual IR of DHF has increased significantly over the last five decades [7,8], molecular epidemiology data are lacking in some regions of Indonesia. Most molecular epidemiology studies were conducted in cities on Java Island including Jakarta [9], Semarang [10], Sukabumi [11], Surabaya [12,13,14,15,16], and Purwokerto [17] and Bali [18,19], with limited studies outside Java and Bali.

Until 2014, there was no published data of circulating DENV in the Aceh and Jambi provinces of Indonesia. Aceh was the area most severely affected by the earthquake and tsunami of 26 December 2004. Data over the 2003 to 2011 period revealed an upward trend of dengue cases in Aceh from 2.76 per 100,000 in 2003 to 56.40 per 100,000 in 2011 [20]. Although the number of dengue cases has increased in this region, molecular epidemiology data are not available. In addition, the currently available dengue vaccine highlights the need for data on current and previous circulating DENV serotypes, genotypes, and lineages in target populations, such as in Indonesia. Therefore, more molecular epidemiology data are needed, particularly from regions with limited available data.

In Jambi, another province located on Sumatra Island of Indonesia, a significant increase of DHF cases was also observed during 2014, from 638 cases in 2013 which doubled to 1308 in 2014 [21,22]. A molecular epidemiology study was conducted in Jambi to assess the circulating DENV associated with the outbreaks during 2014–2015 [23] and found that all four DENV serotypes co-circulated during the outbreaks and that DENV-1 Genotype I was the dominant strain [23]. However, as there were no previous molecular studies conducted in Jambi prior to 2014, the results are difficult to interpret with regard to transmission dynamics. Whether viruses associated with the outbreaks evolved locally or were imported from other locations within Indonesia, or from overseas, is not known. Further studies are needed to assess the DENV transmission dynamics in Jambi, to assess possible introductions, shifts or replacements of DENV serotypes, genotypes or lineages.

Chikungunya virus (CHIKV) is also endemic in Indonesia. The first chikungunya outbreak was reported in 1982 [24] and re-emerged in early 2001, with multiple outbreaks reported subsequently [25]. Although there has been an increase in reported chikungunya cases in the country, molecular epidemiology studies are limited with most studies being conducted in Java [26,27,28,29]. During the 2014–2015 dengue outbreaks in Jambi [23], molecular epidemiology studies were conducted to identify circulating CHIKV as well as other arboviruses [30,31]. These studies identified the co-circulation of DENV, CHIKV and Zika virus (ZIKV) [30,31]. Given that Aceh and Jambi are in close proximity in Sumatra, it is also important to determine the presence and circulation dynamics of DENV and CHIKV in both areas and to clarify the circulation dynamics between the two provinces. This is also important with regard to public health as co-circulation of arboviruses is likely to lead to misdiagnoses and underreporting of infections. This study sought to determine the serotypes, genotype, lineages and phylogenetic relationship of DENV and CHIKV circulating in western parts of Indonesia, in Aceh and Jambi province.

## 2. Materials and Methods

### 2.1. Study Setting and Patient Recruitment

A year-long, cross-sectional study was conducted in Aceh (2015) and Jambi (2016). Probable dengue cases were recruited and serum samples were collected. In Aceh, patient recruitment was conducted in four hospitals in the capital city of Banda Aceh (Dr. Zainoel Abidin Hospital, Meuraxa Hospital, Kesdam Hospital and Ibu Anak Hospital). In Jambi, the patients were recruited in Siloam Hospital, which is located in the capital city of the province.

All patients (adult and children) with probable dengue based on WHO criteria [32] were invited to participate and provided informed consent upon enrolment into the study. Probable diagnostic criteria include acute febrile illness with two or more conditions including headache, retro-orbital pain, myalgia, arthralgia, rash, hemorrhagic manifestations, leucopoenia, thrombocytopenia, rise of hematocrit level, and at least one of following conditions: (a) supportive serology on a single serum sample and (b) occurrence at the same location and time as confirmed cases of DF [32].

Demographic data, clinical manifestations and laboratory information from all probable dengue patients were collected. On the first visit day, approximately 3–5 mL of venous blood was collected, and serum samples were sent to Eijkman Institute for Molecular Biology in Jakarta and stored at −80 °C before use.

### 2.2. NS1 EIA and DENV-Serotyping

Serum samples were initially tested for the presence of DENV NSI antigen with Platelia Dengue NS1 Antigen EIA kit (Bio-Rad, Marnes-la-Coquette, France). To attempt DENV isolation, NS1-positive sera were inoculated and cultured on to Vero cells (African Green Monkey kidney epithelium, ATCC CCL-81) maintained in Dulbecco’s Modified Eagle Media (Thermo Fisher Scientific, Waltham, MA, USA) supplemented with 5% heat-inactivated fetal bovine serum, 1% l-glutamine and 1% penicillin/streptomycin (Life Technologies, Carlsbad, CA, USA). Cultures were incubated at 37 °C with 5% CO_2_ for 7 days and blind passaged for two rounds to increase the isolation success rate. NS1-negative samples were also inoculated onto Vero cells to attempt isolation of other viruses. All NS1-positive samples were subjected to DENV serotyping PCR using an in-house designed RT-PCR targeting the NS5 gene region (PathWest Laboratory, Nedlands, WA, Australia).

### 2.3. DENV E Genes Amplification and DENV E Gene Sequencing

DENV RNA was extracted from culture supernatant using the spin protocol of the QIAamp Viral RNA Mini kit (Qiagen, Hilden, Germany). Complementary DNA was synthesized using the SuperScript III First-Strand Synthesis System for RT-PCR following the manufacturers’ protocol (Invitrogen—Life Technologies, Carlsbad, CA, USA). Two pairs of specific primers for each serotype were used concurrently to amplify the E gene, the primer sequences for which have been published elsewhere [33]. The amplifications were run using a PCR master mix GeneAmp 10× PCR buffer II and Taq DNA polymerase (AmpliTaq Gold 360 DNA Polymerase, Applied Biosystems, Foster City, CA, USA) with 45 and 40 cycles for DENV E and CHIKV E1, respectively. The size of the PCR product fragments varied among serotypes: DENV1: 840 and 1182 bp; DENV-2: 1031 and 990 bp; DENV-3: 1528 and 865 bp; DENV-4: 1096 and 970 bp.

Gene sequencing was conducted using the Sanger sequencing approach [34]. Prior to sequencing, the excess oligonucleotide primers and dNTPs from PCR product were removed using Illustra ExoStar (GE Healthcare Life Sciences, Buckinghamshire, UK). The BigDye Terminator v3.1 Cycle Sequencing kit and BigDye Terminator 5× Sequencing Buffer (both from Applied Biosystem, Foster City, CA, USA) were used for the DNA sequencing reaction. The samples were re-cleaned using the DyeEx 2.0 Spin kit (Qiagen GmbH, Hilden, Germany) and a capillary electrophoresis was performed on a 16-capilary genetic analyzer (3130xl Genetic Analyzer, Applied Biosystems, Foster City, CA, USA).

### 2.4. CHIKV Whole Genome Sequencing

Whole CHIKV genomes were determined by Illumina sequencing. Vero cell virus culture supernatant was harvested and then concentrated using Millipore centrifugal filter unit (100,000 kDa) for 20 min at 40,000× *g* (Merck Millipore Ltd., County Cork, Ireland). Total RNA was extracted from 150–200 μL of concentrated virus using the Roche High Pure RNA Isolation kit as per manufacturers’ instructions (Roche Diagnostic GmbH, Mannheim, Germany). DNA libraries were prepared with the Illumina TruSeq Stranded mRNA kit (Illumina Inc., San Diego, CA, USA). The DNA library was validated with a High Sensitivity DNA kit on the Agilent 2100 Bioanalyzer (both from Agilent Technologies, Waldbronn, Germany). The concentration of the libraries was normalized to 4 nM using HT1 buffer and pooled together. The final concentration of the pooled DNA library was then re-examined using the Qubit dsDNA Assay kit (Invitrogen—Life Technologies, Carlsbad, CA, USA). The pooled DNA library was sequenced using a MiSeq Reagent Micro Kit, v2 (Illumina Inc., San Diego, CA, USA). The high throughput sequence reads from WGS were paired and used to construct contiguous sequences, de novo, using CLC Genomics Workbench 10.0 (Qiagen, Aarhus, Denmark) after quality assessment within FastQC Version 0.11 (Babraham Bioinformatics, Cambridge, UK).

### 2.5. Phylogenetic Analysis

For each virus, derived sequences (DENV E gene and CHIKV complete genomes) together with available sequences from GenBank were aligned separately using Multiple Alignment using Fast Fourier Transform (MAFFT) v.7.309 [35] as implemented in Geneious v.10.1.3 [36]. To provide the relationship between Indonesian viruses and viruses isolated from other parts of world, an estimation of the maximum likelihood-based phylogenetic tree was performed for each virus using RAxML v.7.2.8 [37,38]. GTR with gamma substitution model (GTR+Γ) and a rapid bootstrap procedure (100 replicates) were employed. Resultant phylogenies were visualized using FigTree v1.4.3 and bootstrap of at least 80% was used as an indicator for strong support for grouping of viruses into distinct lineages.

## 3. Results

### 3.1. Patient Characteristics

In total, 205 dengue-suspected patients were recruited (63 from Aceh and 142 from Jambi), of which 56 were confirmed as dengue based on NS1 EIA (29/63 from Aceh and 27/142 from Jambi). Among the 56 confirmed dengue cases, the median age was 21.3 years with the majority aged between 21–30 years (33.9%), followed by the 11–20 years age group (26.8%) (Table 1). There was an equal number of reported male and female cases. Complete clinical histories were available for 43 patients from both regions. The most common reported clinical symptoms were fever, nausea or vomiting, headache, and aches and pains (myalgia or arthralgia). Dengue with warning signs based on the WHO criteria [39] was observed in 12 (27.9%) patients in whom abdominal pain or tenderness was the most frequent sign (20.9%) followed by mucosal bleeding (9.3%). Of those with a warning sign, the vast majority (11, 91.6%) were from Aceh, most from the Zainoel Abidin Hospital (8, 66.6%). This is unsurprising, given the Zainoel Abidin Hospital is a provincial referral hospital where most of the severe cases were referred. No severe dengue was identified in this study.

### 3.2. Serotype Distributions of DENV in Aceh and Jambi

**Figure 1:**
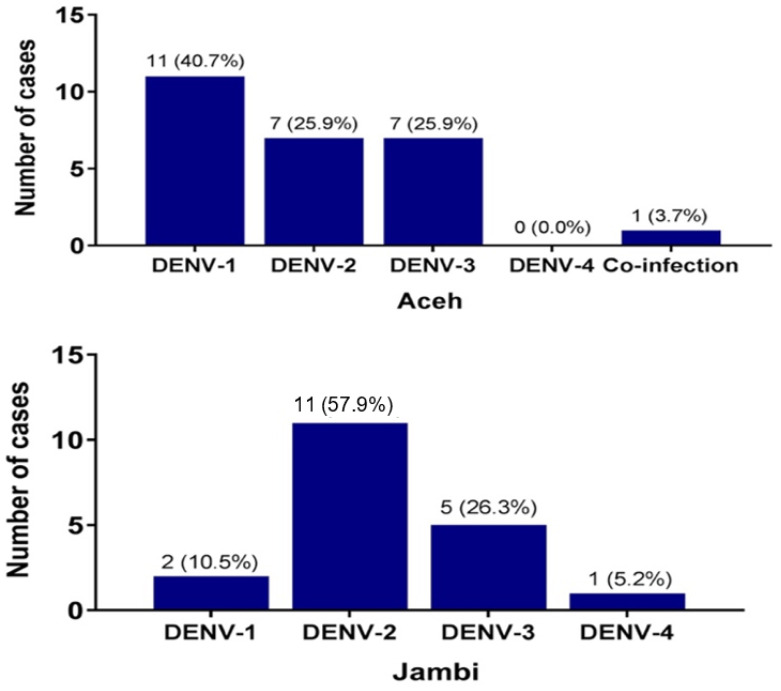
Serotype distribution of DENV isolated from Aceh (2015) and Jambi (2016). In Aceh, there was one co-infection of two serotypes (DENV-2 and DENV-3).

Twenty six of 29 (89.6%) NS1 positive samples from Aceh were successfully serotyped by PCR. DENV-1 was the dominant serotype (11/27, 40.7%); DENV-2 and DENV-3 were detected in equal number (7/27, 25.9%) and no DENV-4 was identified (Figure 1). One co-infection of two serotypes (DENV-2 and DENV-3) was observed. In Jambi, 19 of 27 (70.3%) NS1 positive serum samples were successfully serotyped. All four DENV serotypes were found to circulate in Jambi in 2016, with DENV-2 as the most dominant (11/19, 57.8%), followed by DENV-3 (5/19, 26.3%), DENV-1 (2/19, 10.5%) and DENV-4 (1/19, 5.2%) (Figure 1).

### 3.3. Distribution of DENV Genotypes and Lineages in Aceh and Jambi

A total of eight DENV sequences from Aceh were successfully sequenced of which, five were typed as DENV-1 and three as DENV-2 (Table 2). All characterized DENV-1 viruses were genotyped as Genotype I and all DENV-2 viruses belonged to the Cosmopolitan genotype. In Jambi, 14 E genes were sequenced (2 DENV-1, 8 DENV-2 and 4 DENV-3). Similar to what was observed in Aceh, all DENV-1 and DENV-2 viruses grouped within the Genotype I and Cosmopolitan genotypes, respectively. Phylogenetic analysis revealed that all DENV-3 belonged to Genotype I (Table 2).

#### 3.3.1. DENV-1

**Figure 2:**
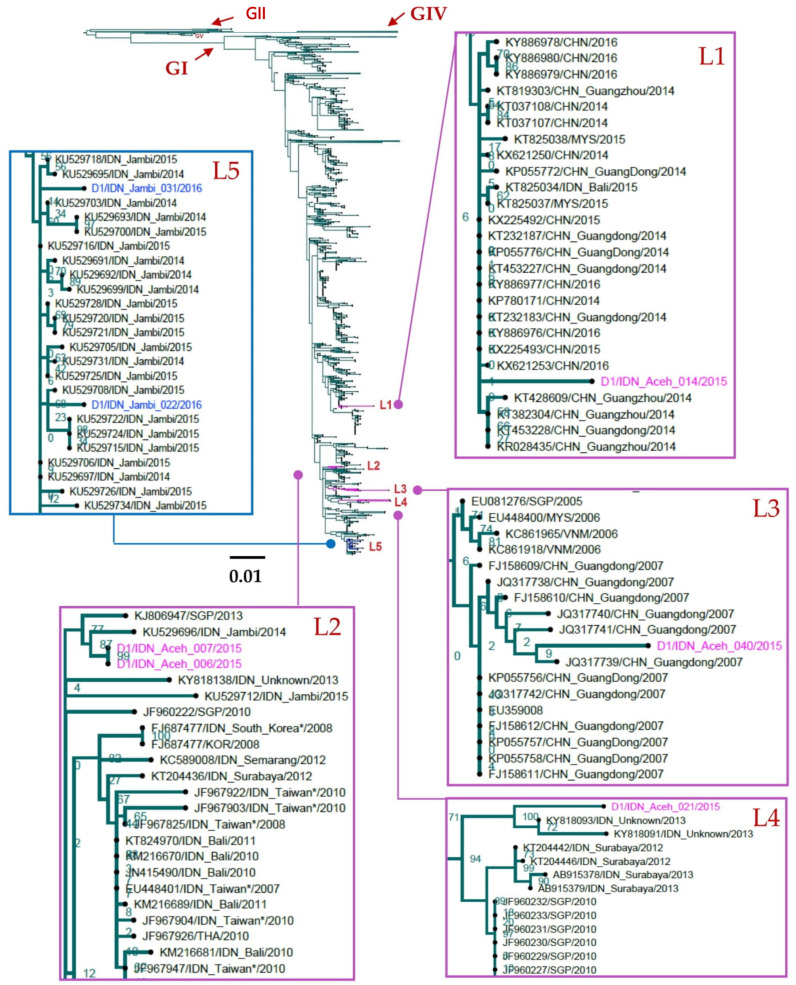
Phylogenetic tree of DENV-1 isolated from Aceh and Jambi showing Lineages 1–5. The phylogenetic tree was generated using the maximum likelihood method using RAxML with a GTR+Γ model with 4268 sequences from NCBI. Some phylogenetic clusters have been collapsed to reveal the positions of isolated viruses in more detail. Pink indicates viruses isolated from Aceh and blue indicates viruses isolated from Jambi in this study. G: genotype, L: lineage. CHN: China, IDN: Indonesia, SGP: Singapore, THA: Thailand. Asterix (*) indicates virus isolated from travelers returning from Indonesia.

All DENV-1 isolated from Aceh and Jambi belong to DENV-1, Genotype I (Figure 2). Viruses from Aceh fell into four phylogenetically distinct lineages (Lineage 1–4) and clustered with sequences from the Southeast Asia region. These lineages could be further differentiated into two major groups based on the origin of the viruses: Indonesian local lineages (Lineage 2 and 4) and introduced lineages (Lineage 1 and 3) (Figure 2).

Lineage 1 consisted of one virus from Aceh (D1/IDN_Aceh_014/2015). Interestingly, most sampled viruses of this lineage were viruses isolated from China during 2014–2016 and Malaysia in 2014 (Figure 2). This lineage had not been sampled in Indonesia prior to 2015. Viruses of Lineage 2 (D1/IDN_Aceh_007/2015 and D1/IDN_Aceh_006/2016), however, clustered with previously characterized Indonesian viruses including those isolated from Jambi and other parts of Indonesia (Figure 2). These newly characterized Lineage 2 viruses were closely phylogenetically related to a virus isolated from Jambi in 2014 (KU529696) (Figure 2).

A virus within Lineage 3 was closely related to viruses isolated from China in 2007 as well as viruses isolated from Singapore, Malaysia, and Vietnam during 2005–2006 (Figure 2). The Lineage 4 virus (D1/IDN_Aceh_021/2016) was closely related to Indonesian viruses that were isolated in 2013 during phase III trials of the CYD tetravalent dengue vaccine (KY818093 and KY818091) (Figure 2).

Unlike viruses isolated in Aceh, all 2016 Jambi isolates belonged to a single lineage, Lineage 5, and clustered with viruses isolated from Jambi during the 2014–2015 outbreak, indicating ongoing circulation.

#### 3.3.2. DENV-2

**Figure 3:**
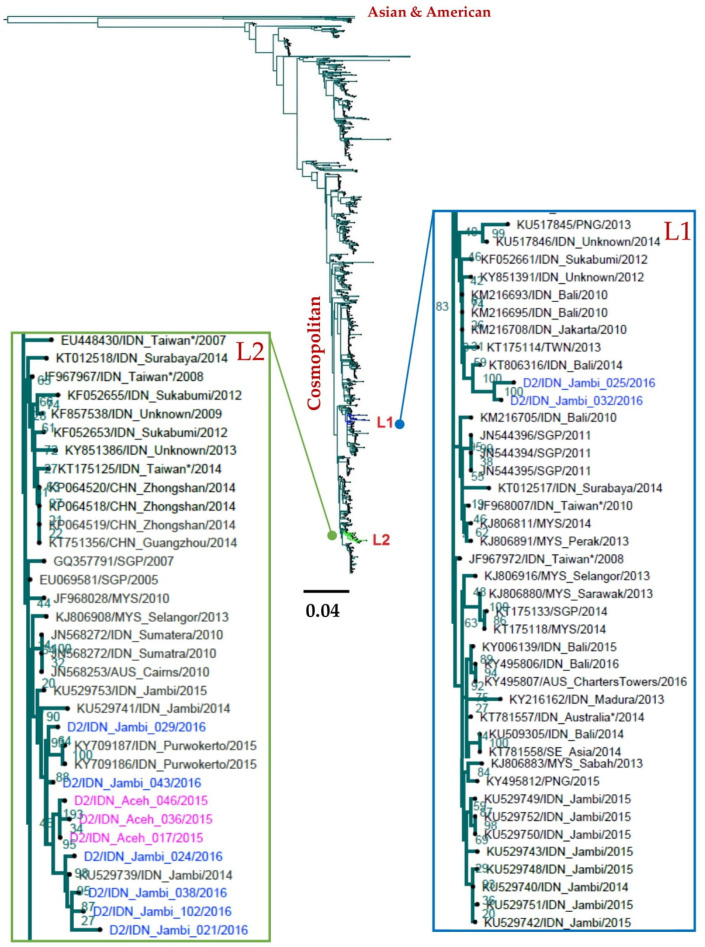
Phylogenetic tree of DENV-2 isolated from Aceh and Jambi showing two lineages (L1 and 2). The tree was generated using the maximum likelihood method using RAxML with a GTR+Γ model of an alignment including three viruses from Aceh, eight viruses from Jambi, and 3469 sequences from NCBI. Some phylogenetic clusters have been collapsed to reveal the positions of isolated viruses in more detail. All identified viruses belong to the Cosmopolitan genotype and clustered into two lineages (L1 and L2). Blue indicates viruses from Jambi and pink indicates viruses isolated from Aceh. Green box indicates the lineage that consists of viruses isolated both from Aceh and Jambi. L: lineage. AUS: Australia, CHN: China, IDN: Indonesia, PNG: Papua New Guinea, MYS: Malaysia, SGP: Singapore, THA: Thailand. Asterix (*) indicates virus isolated from travelers returning from Indonesia.

The three DENV-2 viruses isolated from Aceh and eight from Jambi all belonged to the Cosmopolitan genotype and were further differentiated into two phylogenetically distinct lineages (Lineage 1 and 2) (Figure 3). Lineage 1 consisted of viruses exclusively from Jambi while Lineage 2 consisted of viruses from both regions.

Viruses within Lineage 1 (D2/IDN_Jambi_025/2016 and D2/IDN_Jambi_032/2016) clustered together with viruses isolated from other Indonesian regions Bali, Jakarta, Sukabumi, and from other countries including Papua New Guinea (PNG) (Figure 3). Interestingly, while these viruses were positioned in close relation to the most predominant DENV-2, isolated during the 2014–2015 dengue outbreak in Jambi (Figure 3), the bootstrap support between these two groups of viruses was 83% (well above the threshold of 80%) and therefore could classified into two different lineages.

Lineage 2 included all three DENV-2 sampled from Aceh and six viruses from Jambi. The lineage also included viruses isolated during the 2014–2015 dengue outbreaks in Jambi as well as other viruses from Sumatra and Java such as Purwokerto. This suggests that viruses within this lineage are distributed from the westernmost part of Sumatra Island to Java Island. Interestingly, viruses of this lineage were also isolated from neighboring countries including Singapore and Malaysia.

#### 3.3.3. DENV-3

**Figure 4:**
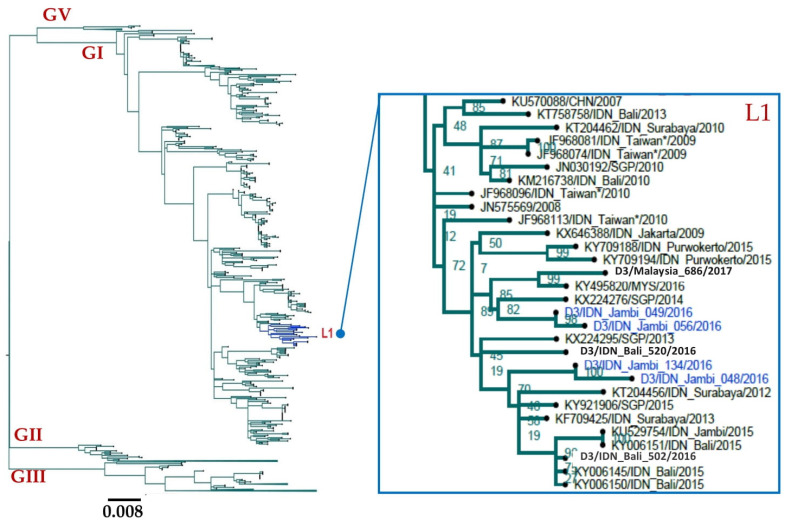
Phylogenetic tree of DENV-3 isolated from Jambi, 2016. The phylogenetic tree was generated using the maximum likelihood method using RAxML with a GTR+Γ model of an alignment including four viruses from Jambi and 2032 sequences from NCBI. Some phylogenetic clusters have been collapsed to reveal the positions of isolated viruses in more detail. All identified viruses belong to Genotype I and clustered into one lineage (L1). Blue indicates viruses isolated from Jambi. G: genotype, L: lineage. CHN: China, IDN: Indonesia, MYS: Malaysia. Asterix (*) indicates virus isolated from travelers returning from Indonesia.

Four DENV-3 viruses were isolated from Jambi and identified as Genotype I (Figure 4). All these viruses grouped into one lineage together with Indonesian strains that were isolated from various locations including Bali, Surabaya, Purwokerto, and Jakarta. This lineage also included the only virus that was isolated (KU529754) during the 2015 dengue outbreak in Jambi [23]. This suggests there was little change in circulating DENV-3 in Jambi between 2015 and 2016. This lineage also contained viruses isolated in Malaysia and Singapore.

### 3.4. Currently Circulating CHIKV in Aceh and Jambi

**Figure 5:**
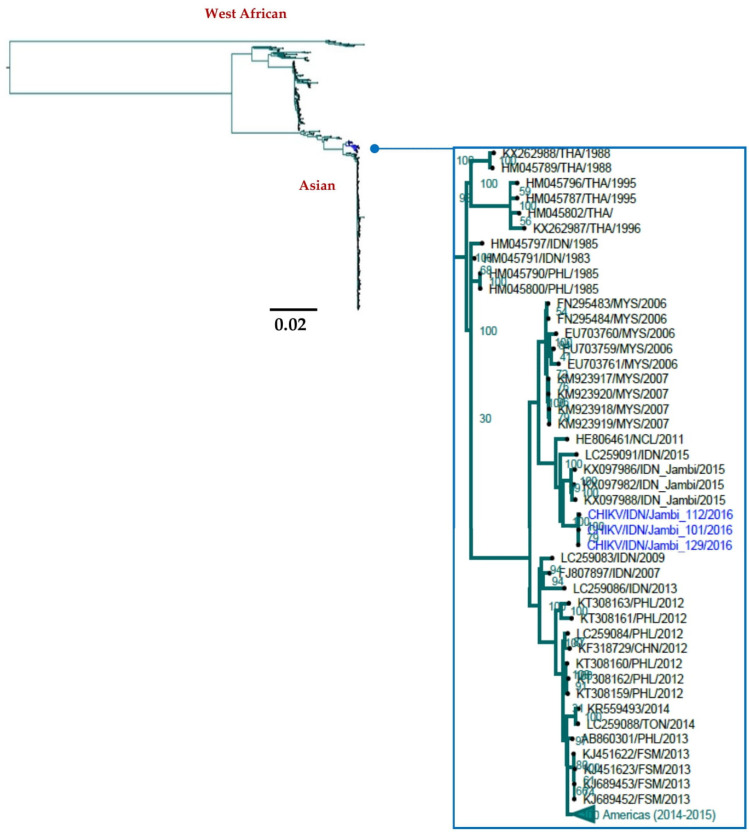
Genome-based phylogeny of CHIKV isolated from Jambi, 2016. The phylogenetic tree was generated using the maximum likelihood method employing RAxML with a GTR+Γ model of an alignment of three CHIKV isolated in Jambi in 2016, 560 sequences from NCBI. Some phylogenetic clusters have been collapsed (indicated as green) to reveal the positions of the viruses from Jambi. CHIKVs sampled from Jambi are colored in blue. CHN: China, FSM: Federated States of Micronesia, IDN: Indonesia, PHL: the Philippine, MYS: Malaysia, NCL: New Caledonia, TON: Tonga, THA: Thailand.

Of 149 NS1-negative sera (34 from Aceh and 115 from Jambi), three were positive for CHIKV as confirmed by RT-PCR (Table 3). Phylogenetic analysis based on full or partial genome sequences indicated these viruses were closely related to viruses that circulated in Jambi in the previous year (2015) (Figure 5). The viruses were also closely related to an Indonesian virus imported to Japan in 2015 (LC259091).

## 4. Discussion

We characterized DENV and CHIKV circulating in Aceh and Jambi, two provinces in western Indonesia, in 2015–2016. This study was the first virological investigation of dengue and chikungunya in Aceh, the western-most region of Indonesia. In Jambi, where an assessment of DENV and CHIKV molecular epidemiology had been conducted in 2014–2015 [23,31], this present follow-up study provides updated and more detailed information on transmission dynamics of both DENV and CHIKV in the region.

### 4.1. Distinct DENV Lineages Circulate in Aceh and Jambi

**Figure 6:**
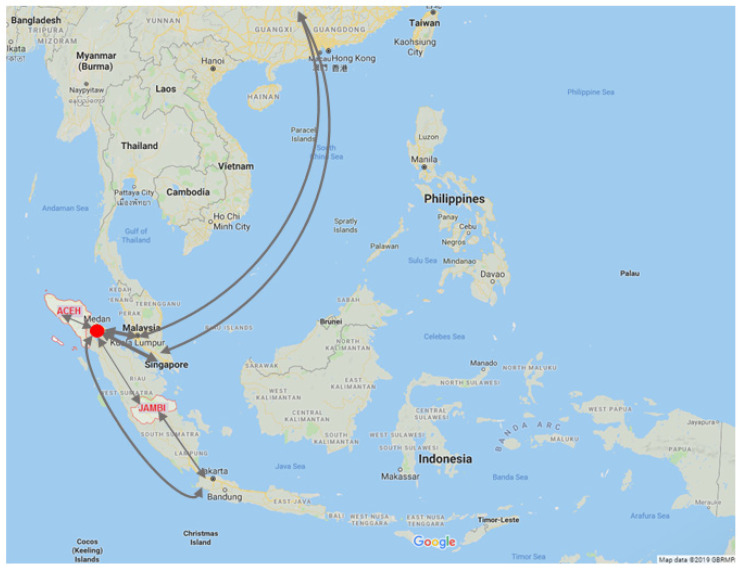
Flight patterns between Aceh and Jambi. The arrows indicate the main flight routes between Aceh, Jambi and neighbor localities. Red dot indicates location of Medan, the capital of North Sumatra Province. Medan is the largest city in Sumatra Island and has major international flight routes connected to neighboring dengue endemic areas, especially Malaysia and Singapore. There is no direct flight between Aceh and Jambi Province.

The molecular epidemiology of DENV in Aceh (2015) and Jambi (2016) was investigated. Although both regions are within Sumatra Island, distinct DENV lineage distributions were observed. Of four DENV-1 Genotype I lineages characterized in Aceh, two (Lineage 1 and Lineage 3) were related to viruses from China and only one (Lineage 2) was closely related and shared a common ancestor with viruses from Jambi (Figure 2). However, the short length E gene sequence fragments from some DENV Lineage 1 and 3 viruses (227 bp), may not have conferred robust phylogenetic reconstruction. Nevertheless, there are some reasons to support the close relationship of viruses between Aceh and China. Aceh Province is located next to North Sumatra Province where Chinese is the fourth most populous ethnicity, most living in Medan, the capital city North Sumatra, with a high population mobility. There are frequent flights between Medan and cities in China via Kuala Lumpur (Malaysia) and Singapore (Figure 6). In addition, there are direct flights between Aceh’s capital, Banda Aceh, and Penang in Malaysia. This transport pattern supports virus movement between Aceh in Indonesia, and China. However, no E gene sequence are available from North Sumatra; the only available sequences are of four NS1 gene sequences (2 DENV-2, 1 DENV-3 and 1 DENV-4) [40]. A brief analysis using Dengue Genographic Viewer [41] indicated, that except for DENV-3, all viruses isolated from North Sumatra Province are closely related with viruses isolated in China.

In contrast, all DENV-1 isolated from Jambi in 2016, grouped within Lineage 5, were closely related to viruses that circulated in Jambi in prior years (2014–2015) (Figure 2). All viruses within Lineage 5 were associated with large-scale dengue outbreaks in Jambi in 2014–2015 in which dengue shock syndrome occurred in 2% of 107 confirmed dengue patients [23]. Interestingly, no DENV-1 isolated from Aceh belong to this lineage indicating distinct DENV-1 viruses circulated in Aceh in 2015, which were distinct to viruses circulating in Jambi during 2014–2015. There are no direct flights between Aceh and Jambi, and flights between Medan and Kuala Lumpur in neighboring Malaysia are far more frequent than between Medan and Jambi, within Indonesia. This pattern of population movements supports our data regarding distinct DENV circulation patterns between geographically distinct, yet proximal, areas in Indonesia.

In contrast to the observed DENV-1 epidemiology, DENV-2 and DENV-3 are more homogeneous between Aceh and Jambi. All DENV-2 from Aceh and Jambi grouped into the Cosmopolitan genotype, the most common genotype in Indonesia and neighboring countries. All DENV-2 viruses clustered with locally sampled Indonesian viruses. The same pattern was observed for DENV-3: all isolated viruses from Jambi belonged to Genotype I and clustered with Indonesian viruses including those that circulated during the 2014–2015 dengue outbreaks in Jambi.

### 4.2. Continuous Co-Circulation of CHIKV and DENV in Jambi

Although multiple chikungunya outbreaks have been reported in Indonesia [25], CHIKV molecular epidemiology studies on Sumatra Island are limited. In the present study based in Aceh (2015) and Jambi (2016), CHIKV was identified in Jambi but not in Aceh. Phylogenetic analysis of CHIKV whole genomes showed that all three Jambi viruses were of the Asian genotype and clustered with Indonesian CHIKV viruses sampled over a wide geographical range including as Sumatra, Java, and Bali, including viruses that circulated in Jambi in 2015 (Figure 5).

This study confirmed continuous co-circulation of DENV and CHIKV in Jambi as reported previously [31]. Together, these data provide insight that DENV and CHIKV co-circulation likely occurs in other areas of Indonesia, a pattern that has been reported by others [29,42]. Continuous and sustainable transmission of CHIKV in Jambi could add the burden of dengue management in particular owing to the absence of confirmatory laboratory testing for CHIKV in most hospitals in Indonesia. Our findings emphasize the importance of laboratory testing for CHIKV among dengue-suspected patients.

The lack of confirmatory laboratory testing for CHIKV and the similarity of dengue and chikungunya clinical presentations may contribute to under-estimation of chikungunya incidence, disease and mortality. No chikungunya-associated deaths have been recorded in Indonesia since the disease was officially recognized by Indonesian Ministry of Health (MoH) in 1973 [43].

Since 2004, chikungunya has been included in the MOH National Diseases Surveillance Program, and since 2005, reported in the Annual Report of the Indonesia Health Profile, the formal annual report of notifiable diseases from MoH Indonesia [43]. In the National Guideline of Prevention and Control of Chikungunya from MoH [44], there are no clear criteria for assessing or reporting chikungunya-related deaths that should be included in the surveillance system. In addition, as chikungunya shares some clinical signs with dengue and not all reported dengue cases in Indonesia are confirmed by laboratory testing, there is a possibility that chikungunya-related deaths may be wrongly attributed to dengue as a consequence of the prioritization of dengue surveillance. Chikungunya is not considered a priority disease in Indonesia, there is poor surveillance and the disease is underreported. For example, chikungunya was excluded from the 2016 MoH Annual Report of the Indonesia Health Profile [45] and was included in the 2017 Annual Report [46]. Our study confirms that CHIKV circulated in Indonesia during 2016.

It would be beneficial if MoH would institute a clear reporting system for chikungunya and provide clear criteria for chikungunya-associated deaths in the national guidelines. In addition, during disease outbreaks active surveillance may be required to reduce underreporting of chikungunya deaths in the country.

## 5. Conclusions

Our phylogenetic analysis showed that all DENV-1 and DENV-2 viruses from Aceh and Jambi belonged to the Genotype I and Cosmopolitan genotypes, respectively and further, that all DENV-3 viruses from Jambi were of the Genotype I. Some viruses, particularly DENV-1, displayed distinct lineage distribution, where two distinct DENV-1 lineages from Aceh contained viruses more closely related to viruses from China, than to Jambi isolates. In contrast, DENV-2 from both regions and DENV-3 from Jambi were all closely related to local Indonesian viruses. Phylogenetic analyses of CHIKV genome sequences from Jambi showed that all viruses belonged to the Asian genotype. These viruses clustered closely with Indonesian CHIKV viruses including those previously circulating in Jambi in 2015—confirming continuous and sustainable transmission of CHIKV in the region. These data emphasize the importance of simultaneous testing for CHIKV among dengue-suspect patients.

## Figures and Tables

**Table 1:** Demographic and clinical characteristics of dengue cases in Aceh and Jambi.

Characteristics	Aceh (*n =* 29) *n* (%)	Jambi (*n =* 27) *n* (%)	Total *n* (%)
**Gender**			
Male	13 (44.8)	15 (55.6)	28 (50.0)
Female	16 (55.2)	12 (44.4)	28 (50.0)
**Age (year)**			
0–10	3 (10.3)	9 (33.3)	12 (21.4)
11–20	12 (41.4)	3 (11.1)	15 (26.8)
21–30	12 (41.4)	7 (25.9)	19 (33.9)
31–40	2 (6.9)	5 (18.5)	7 (12.5)
41–50	0 (0.0)	2 (7.4)	2 (3.6)
>50	0 (0.0)	1 (3.7)	1 (1.8)
**Clinical symptoms (*n* = 43)**	***n*** **= 16**	***n*** **= 26**	
Fever	16 (100.0)	27 (100.0)	43 (100.0)
Nausea or vomiting	15 (93.8)	27 (100.0)	42 (97.7)
Aches and pains	15 (93.8)	12 (44.4)	27 (62.8)
Retro-orbital pain	10 (62.5)	4 (14.8)	14 (32.6)
Headache	13 (81.3)	25 (92.6)	38 (88.4)
**Warming signs (*n* = 43)**			
Abdominal pain or tenderness	8 (50.5)	1 (3.7)	9 (20.9)
Persistent vomiting	0 (0.0)	0 (0.0)	0 (0.0)
Clinical fluid accumulation	0 (0.0)	0 (0.0)	0 (0.0)
Mucosal bleeding	4 (25.5)	0 (0.0)	4 (9.3)
Lethargy	0 (0.0)	0 (0.0)	0 (0.0)
Liver enlargement	2 (12.5)	0 (0.0)	2 (4.7)
**Severe dengue signs (*n* = 43)**			
Respiratory distress	0 (0.0)	0 (0.0)	0 (0.0)
Severe bleeding	0 (0.0)	0 (0.0)	0 (0.0)
Impaired consciousness	0 (0.0)	0 (0.0)	0 (0.0)

**Table 2:** List of DENV isolates from Aceh and Jambi and their phylogenetic characterization.

Origin	Sex	Age (Year)	Collection Date (D/M/Y)	Isolate ID	Serotype	Genotype	Lineage	E-Gene Sequence
Aceh	Female	53	27/03/2015	D1/IDN_Aceh_006/2015	DENV-1	I	L2	Complete
Aceh	Female	21	14/09/2015	D1/IDN_Aceh_007/2015	DENV-1	I	L2	Complete
Aceh	Male	19	12/12/2015	D1/IDN_Aceh_014/2015	DENV-1	I	L1	Partial (227 bp)
Aceh	Female	11	21/09/2015	D1/IDN_Aceh_021/2015	DENV-1	I	L4	Complete
Aceh	Female	18	29/09/2015	D1/IDN_Aceh_040/2015	DENV-1	I	L3	Partial (227 bp)
Aceh	Male	17	14/09/2015	D2/IDN_Aceh_017/2015	DENV-2	Cosmopolitan	L2	Complete
Aceh	Female	27	19/08/2015	D2/IDN_Aceh_036/2015	DENV-2	Cosmopolitan	L2	Complete
Aceh	Female	20	16/06/2015	D2/IDN_Aceh_046/2015	DENV-2	Cosmopolitan	L2	Complete
Jambi	Female	6	07/11/2016	D1/IDN_Jambi_022/2016	DENV-1	I	L5	Complete
Jambi	Male	33	08/08/2016	D1/IDN_Jambi_031/2016	DENV-1	I	L5	Complete
Jambi	Male	6	30/06/2016	D2/IDN_Jambi_021/2016	DENV-2	Cosmopolitan	L2	Complete
Jambi	Male	7	22/07/2016	D2/IDN_Jambi_024/2016	DENV-2	Cosmopolitan	L2	Complete
Jambi	Female	35	22/12/2016	D2/IDN_Jambi_025/2016	DENV-2	Cosmopolitan	L1	Complete
Jambi	Female	43	08/08/2016	D2/IDN_Jambi_029/2016	DENV-2	Cosmopolitan	L2	Complete
Jambi	Male	24	11/08/2016	D2/IDN_Jambi_032/2016	DENV-2	Cosmopolitan	L1	Complete
Jambi	Male	38	24/08/2016	D2/IDN_Jambi_038/2016	DENV-2	Cosmopolitan	L2	Complete
Jambi	Male	23	16/09/2016	D2/IDN_Jambi_043/2016	DENV-2	Cosmopolitan	L2	Complete
Jambi	Female	30	21/11/2016	D2/IDN_Jambi_102/2016	DENV-2	Cosmopolitan	L2	Complete
Jambi	Male	17	25/09/2016	D3/IDN_Jambi_048/2016	DENV-3	I	L1	Complete
Jambi	Female	9	25/09/2016	D3/IDN_Jambi_049/2016	DENV-3	I	L1	Partial (663 bp)
Jambi	Female	3	07/10/2016	D3/IDN_Jambi_056/2016	DENV-3	I	L1	Complete
Jambi	Male	21	05/12/2016	D3/IDN_Jambi_134/2016	DENV-3	I	L1	Complete

**Table 3:** List of samples tested positive for CHIKV from Jambi.

Sex	Age (Year)	Collection Date (D/M/Y)	ID	RT-PCR CHIKV	Genotype	Sequence
Male	53	21/11/2016	CHIKV/IDN_Jambi_101/2016	Positive	Asian	Full genome
Female	10	24/11/2016	CHIKV/IDN_Jambi_112/2016	Positive	Asian	Full genome
Female	29	02/12/2016	CHIKV/IDN_Jambi_129/2016	Positive	Asian	Partial genome

## Data Availability

DENV and CHIKV sequences were deposited in the GenBank repository with accession numbers OK655882-OK655884 (CHIKV); OK631778-OK631884 (DENV-1); OL960211-OL960221 (DENV-2); OL960230-OL960233 (DENV-3).
